# The *XRCC 1* DNA repair gene modifies the environmental risk of stomach cancer: a hospital-based matched case-control study

**DOI:** 10.1186/s12885-017-3675-9

**Published:** 2017-10-11

**Authors:** Nuntiput Putthanachote, Supannee Promthet, Cameron Hurst, Krittika Suwanrungruang, Peechanika Chopjitt, Surapon Wiangnon, Sam Li-Sheng Chen, Amy Ming-Fang Yen, Tony Hsiu-Hsi Chen

**Affiliations:** 1 Clinical Microbiology Laboratory, Roi-Et Hospital, Roi-Et Province, Thailand; 2 Department of Epidemiology and Biostatistics, Faculty of Public Health, Khon Kaen University, Khon Kaen Province, Thailand; 3 ASEAN Cancer Epidemiology and Prevention Research Group, Khon Kaen University, Khon Kaen Province, Thailand; 4 Center of Excellence in Biostatistics, Faculty of Medicine, Chulalongkorn University, Bangkok, Thailand; 5 Cancer Unit, Faculty of Medicine, Khon Kaen University, Khon Kaen Province, Thailand; 6 Faculty of Public Health, Kasetsart University Chalermphrakiat, Sakon Nakhon Campus, Sakon Nakhon Province, Thailand; 7 Department of Pediatrics, Faculty of Medicine, Khon Kaen University, Khon Kaen Province, Thailand; 8 College of Oral Medicine, School of Oral Hygiene, Taipei Medical University, Taipei, Taiwan; 9 Institute of Epidemiology and Prevention Medicine, College of Public Health, National Taiwan University, Taipei, Taiwan

**Keywords:** *XRCC1* gene, Vegetable oil, Salt intake, Stomach cancer

## Abstract

**Background:**

Previous studies have found that polymorphisms of the DNA repair gene X-ray repair cross-complementing group 1(*XRCC1*) and environmental factors are both associated with an increased risk of stomach cancer, but no study has reported on the potential additive effect of these factors among Thai people. The aim of this study was to investigate whether the risk of stomach cancer from *XRCC1* gene polymorphisms was modified by environmental factors in the Thai population.

**Methods:**

Hospital-based matched case-control study data were collected from 101 new stomach cancer cases and 202 controls, which were recruited from2002 to 2006 and were matched for gender and age. Genotype analysis was performed using real-time PCR-HRM. The data were analysed by the *chi*-square test and conditional logistic regression.

**Results:**

The Arg/Arg homozygote polymorphism of the *XRCC1* gene was associated with an increased risk of stomach cancer in the Thai population (OR _adj_, 3.7; 95%CI, 1.30–10.72) compared with Gln/Gln homozygosity. The effect of the *XRCC1*gene on the risk of stomach cancer was modified by both a high intake of vegetable oils and salt (*p* = 0.036 and *p* = 0.014), particularly for the Arg/Arg homozygous genotype. There were, however, no additive effects on the risk of stomach cancer between variants of the *XRCC1*gene and smoking,alcohol or pork oil consumption.

**Conclusions:**

The effect of the *XRCC1* gene homozygosity, particularly Arg/Arg, on the risk for stomach cancer was elevated by a high intake of vegetable oils and salt.

## Background

Stomach cancer is the fourth most common type of cancer worldwide and is a leading cause of death; there were an estimated 723,000 deaths in 2012 due to stomach cancer [1]. Previous studies have reported on environmental risk factors that influence stomach cancer incidence, including smoking, high salt intake, *H. pylori* infection and consumption of alcohol, sausages, or foods at hot temperatures [2–8]. Other studies have demonstrated that dietary vegetable oils and consumption of animal fats and processed meat increase the risk of stomach cancer [9–12].

The X-ray repair cross-complementing group 1 (*XRCC1*) gene is a genetic variant that has been widely implicated in cancer susceptibility. Evidence from 297 case-controlled studies found that the *XRCC1* gene increases the overall risk for cancer [13]. More recent work suggests that the *XRCC1* gene is an important risk factor for stomach cancer [14–17].

Numerous studies have investigated interactive effects between gene and environmental risk factors for cancer, finding that the impacts of smoking, alcohol consumption and dietary factors are all modified by genotype [18–23]. While studies have shown the separate contributions of oils consumption, smoking, alcohol intake and the *XRCC1* gene in the development of stomach cancer, little is known about the multiplicative effects of these factors, and there have been no previous studies on the gene-environment interaction in stomach cancer risk for the Thai population. The aim of this study was to investigate the multiplicative effects of the *XRCC1*gene and environmental factors on stomach cancer incidence in the population of Northeastern Thailand.

## Methods

### Demographic characteristics of subjects

This was a hospital-based matched case-control study in which data were collected from 101 new stomach cancer cases and 202 hospital controls admitted for other diseases. The controls were matched for age (±3 years) and gender, and all patients were admitted at the same time and in the same wards as the cancer cases. All cases and controls were recruited from KhonKaen Regional Hospital and Srinagarind Hospital in KhonKaen Province, Northeast Thailand, from 2002 to 2006 and all data collection was conducted by expert-trained nurses. The stomach cancer location distribution was 46.5% antrum, 26.3% unspecified sub site, 17.2% cardia, 7.0% body, 2.0% pylorus and 1% fundus. All cases were histologically confirmed and diagnosed according to the International Classification of Diseases for Oncology Third edition (ICD-O 3rd). Cancer was most commonly in stage IV (53.5%). All cases and controls were in Northeast Thailand (typically Thai-Lao ethnicity) and all subjects gave written informed consent for their participation in the study.

Data on cases and controls were obtained via an interviewer-based structured questionnaire and blood samples collected at the time of recruitment. The factors of interest were demographic information, smoking status, alcohol, oil consumption, and salt intake**.**

Alcohol consumption was separated into two categories (drinkers and non drinkers), with drinkers defined as those who had consumed alcohol (beer, white whisky, red whisky and other whiskies) at least once a month and non drinkers defined as those who consumed alcohol less than once a month. Smokers were those who reported that they had smoked at least one cigarette per day for at least six months prior to diagnosis.

Dietary consumption of vegetable oil, pork oil and salt were categorized as high or low based on consumption frequency, with low levels corresponding to reports of consuming sometimes, rarely or never and high corresponding to consumption often or always.

### Laboratory method

Specimen blood samples were obtained from all 101 cases and 202 controls. Whole blood samples of 3–5 ml were collected after interviews and centrifuged at 3000 rpm for 15 min to separate the plasma, buffy coat and red blood cells. All specimens were stored at −20 °C at the cancer unit, Faculty of Medicine, KhonKaen University, Thailand.

Genomic DNA extractions were obtained from the buffy coat and were analysed at Nagoya city, University Medical School Nagoya, Japan. PCR amplification, genetic polymorphism detection, and genomic DNA extracted from the buffy coat of all participants were analysed using real-time polymerase chain reaction with high resolution melting (Real-time PCR-HRM).

The *XRCC1* Gln399Arg DNA amplification used two primers, [F]: 5′-AGT GGG TGC TGG ACT GTC-3′ and [R]:5′-TTG CCC AGC ACA GGA TAA-3′, and was performed in a lightCycler® 480 Real-Time PCR System. HRM data were analysed using lightCycler® 480 Gene Scanning software version 1.5(Roche) at the microbiology laboratory, Faculty of Medicine, KhonKaen University, Thailand.

Although *H .pylori* infection status of the subjects was investigated at diagnosis, for the cancer patients, it was not recorded at any time for our control participants. For this reason, we did not include *H. pylori* infection as a risk factor in this study.

### Statistical analysis

The general characteristics of subjects were summarized in the form of percentages, means and standard variations, depending on the scale of the variables. Univariate analysis was conducted with McNemar’s *chi*-square to test for Hardy-Weinberg equilibrium. Bivariate multivariable conditional logistic regression modelling was used to obtain unadjusted and adjusted estimates of association between the *XRCC1* gene, smoking, salt intake, and alcohol and oil consumption, and stomach cancer. Statistical significance was set as *p*-value <0.05, and all data analyses were performed using STATA software, version 10.0.

## Results

### Demographic characteristics of samples

The characteristics of the 101 stomach cancer cases and 202 controls are provided in Table 1. The age and gender distributions were similar between cases and controls (Male: 57.4% and 56.4%;Female: 42.6 and 43.6%;mean age 52.7 years; SD = ± 11.42 and 52.7 years; SD = ± 10.00). For both cases and controls, most participants were married, farmers, had graduated from at least primary school and generally exhibited high vegetable oil and salt intake.

**Table 1:** Demographic characteristics of stomach cancer cases and controls

Variables	Cases (%) *n* = 101	Controls (%) *n* = 202	*p-value*
Gender			0.806
Male	57 (56.4)	114 (56.4)	
Female	44 (43.6)	88 (43.6)	
Age (years)			0.730
< 60	70 (69.3)	134 (66.3)	
= > 60	31 (30.7)	68 (33.7)	
Mean +/- SD	52.7 (11.42)	52.7 (11.00)	
Marital status			0.533
Single	6 (5.9)	6 (2.9)	
Married	79 (78.2)	168 (83.2)	
Separated, widowed	16 (15.9)	28 (13.9)	
Occupation			0.927
Agriculture, farmer	70 (69.3)	141 (69.8)	
Office, technical work	18 (17.8)	47 (23.3)	
Professional work	13 (12.9)	14 (6.9)	
Education			0.086
Illiteracy	2 (2.0)	5 (2.5)	
Primary school	75 (74.3)	168 (83.2)	
Secondary school or higher	24 (23.7)	29 (14.3)	
Family history of cancer			0.003
No	61 (60.4)	157 (77.7)	
Yes	40 (39.6)	45 (22.3)	
Gastritis history			0.088
No	46 (45.5)	111 (55.0)	
Yes	55 (54.5)	91 (45.0)	
Smoking			0.416
Nonsmoker	49 (48.5)	107 (53.0)	
Smoker	52 (51.5)	95 (47.0)	
Alcohol drinking			0.123
Nondrinker	46 (45.5)	110 (54.4)	
Drinker	55 (54.5)	92 (45.6)	
Vegetable oil intake			0.094
Low intake	3 (3.0)	17 (8.4)	
High intake	98 (97.0)	185 (91.6)	
Pork oil intake			0.414
Low intake	89 (88.1)	184 (91.1)	
High intake	12 (11.9)	18 (8.9)	
Salt intake			0.066
Low intake	46 (45.5)	72 (35.6)	
High intake	55 (54.5)	130 (64.4)	

### Pathological characteristics of cases

In summarizing the pathological characteristics of the cases (Table 2), the most common specified anatomical sites of stomach cancer were the antrum (46.5%) and cardia (17.2%). In terms of histopathology, the most frequently observed features were signet ring cell carcinoma (24.7%), adenocarcinoma not otherwise specified (69.3%), poly differentiated (58.4%) and unable to be assessed (20.8%). In the majority of patients, cancer was found to be at stage IV (53.5%), but the stage was unknown in 23.8% of patients. The distribution of genotypes did not differ from the expected frequencies under Hardy-Weinberg equilibrium in either the cases (*P* = 0.482) or controls (*P* = 0.361).

**Table 2:** Pathological characteristics of the malignancies in the cases

Pathological	Number (%) n = 101
Site of cancer	
Fundus	1(1.0)
Pylorus	2(2.0)
Body	7(7.0)
Cardia	17(17.2)
Antrum	46(46.5)
Stomach, NOS	28(26.3)
Histology type	
Tubular adenocarcinoma	1(1.0)
Diffuse type	5(5.0)
Signet ring cell carcinoma	25(24.7)
Adenocarcinoma, NOS	70(69.3)
Histology grading	
Well differentiated	10(9.9)
Moderately differentiated	11(11.9)
Poorly differentiated	59(58.4)
Grade cannot be assessed	21(20.8)
Stage of diseases	
Stage IB	3(2.9)
Stage II	5(5.0)
Stage IIIA	9(8.9)
Stage IIIB	6(5.9)
Stage IV	54(53.5)
Unknown stage	24(23.8)

*NOS:* not otherwise specified

### Frequency of variants of the *XRCC1* genotypes and environmental factors and their associations with stomach cancer

The allele frequencies for the *XRCC1* Gln399Arg genotypes in the cases and controls were 47.5 and 54.5% for Gln/Gln, 40.6 and 41.5% for Gln/Arg and 11.9 and 4.0% for Arg/Arg, respectively. Table 3 provides the results of multivariable binary conditional logistic regression analyses, which revealed that the *XRCC1* Gln399Arg genotype, Arg/Arg homozygous, was found to be associated with stomach cancer (OR _adj. =_ 3.7; 95%CI: 1.30–10.72) relative to Gln/Gln homozygous. However, there was no statistically significant association with Gln/Arg (OR _adj._ =1.2; 95%CI: 0.70–1.97) heterozygosity. For the environmental factors and their associations with stomach cancer, statistical significance was found for both a family history of cancer (OR _adj._ =2.0; 95%CI: 1.37–4.00) and high vegetable oil intake (OR _adj._ =3.2; 95%CI: 1.90–11.59). However, there were no significant associations with a history of gastritis, smoking, salt intake, consumption of alcohol or pork oil.

**Table 3:** Crude and adjusted analyses association of genotype and environmental factors with stomach cancer

Variable	Cases (%)	Controls (%)	ORc (95% CI)	ORadj. (95% CI)	*p-value*
	n = 101	n = 202			
*XRCC1 gene*					
Gln/Gln	48 (47.5)	110 (54.5)	1.0	1.0	0.041
Gln/Arg	41 (40.6)	84 (41.5)	0.9 (0.59–1.55)	1.2 (0.70–1.97)	
Arg/Arg	12 (11.9)	8 (4.0)	3.6 (1.32–9.60)	3.7 (1.30–10.72)	
Gender					0.754
Male	57 (56.4)	114 (56.4)	1	1	
Female	44 (43.6)	88 (43.6)	1.1 (0.83–1.49)	1.5 (0.15–22.62)	
Age (years)					0.120
< 60	70 (69.3)	134 (66.3)	1	1	
= > 60	31 (30.7)	68 (33.7)	0.5 (0.15–1.61)	0.3 (0.15–1.58)	
Family history of cancer					0.013
No	61 (60.4)	157 (77.7)	1	1	
Yes	40 (39.6)	45 (22.3)	2.3 (1.36–3.93)	2.0 (1.37–4.00)	
Gastritis history					0.236
No	46 (45.5)	111 (55.0)	1	1	
Yes	55 (54.5)	91 (45.0)	1.4 (0.89–2.31)	1.3 (0.81–2.26)	
Smoking					0.399
Nonsmoker	49 (48.5)	107 (53.0)	1	1	
Smoker	52 (51.5)	95 (47.0)	1.9 (0.76–5.01)	1.6 (0.54–4.48)	
Alcohol drinking					0.140
Nondrinker	46 (45.5)	110 (54.4)	1	1	
Drinker	55 (54.5)	92 (45.6)	1.8 (0.98–3.44)	1.7 (0.84–3.21)	
Vegetable oil intake					0.028
Low intake	3 (3.0)	17 (8.4)	1	1	
High intake	98 (97.0)	185 (91.6)	3.0 (1.85–10.33)	3.2 (1.90–11.59)	
Pork oil intake					0.173
Low intake	89 (88.1)	184 (91.1)	1	1	
High intake	12 (11.9)	18 (8.9)	1.4 (0.62–3.19)	1.9 (0.74–5.13)	
Salt intake					0.124
Low intake	46 (45.5)	72 (35.6)	1	1	
High intake	55 (54.5)	130 (64.4)	0.6 (0.37–1.05)	0.7 (0.37–1.06)	

*ORc:* crude odd ratio, *ORadj.:* adjusted odd ratio, *95% CI*: 95% confidence interval,  *p*-value from conditional logistic regression

### Interaction of environmental factors with the *XRCC1* genotypes and their associations with stomach cancer

We also investigated whether there was an *XRCC1* gene and environmental interaction with each environmental risk factor (Table 4). The analysis revealed that the *XRCC1* Gln399Arg genotype is a significant effect modifier of environmental risk of stomach cancer for both high vegetable oil consumption (*p* = 0.036) and high salt intake (*p* = 0.014). Specifically, a high vegetable oil intake represents a significant risk factor for stomach cancer for an Arg/Arg homozygote genotype (OR _adj._ =3.6; 95% CI: 1.27–10.49), but not for a Glu/Glu homozygote genotype (OR _adj._ =0.3; 95%CI: 0.04–2.96) or Gln/Arg heterozygote genotype (OR _adj._ =1.3; 95%CI: 0.76–2.16). Similarly, high salt intake is a significant risk factor for an Arg/Arg homozygote genotype (OR _adj._ = 5.3; 95% CI: 1.34–21.22) but not a Glu/Glu homozygote genotype (OR _adj._ =0.4; 95%CI: 0.18–1.90) or Gln/Arg heterozygote genotype (OR _adj._ =0.6; 95%CI: 0.26–1.28).

**Table 4:** Interaction between the environmental factors with *XRCC1* Gln339Arg polymorphisms as risk factors for stomach cancer

Variable		Cases n (%)	Controls n (%)	ORadj. (95%CI)	*p-value*
*XRCC1* gene x Vegetable oil intake					0.036
Gln/Gln	Low	1 (1.0)	8 (4.0)	1.0	
	High	47 (46.5)	102 (50.5)	0.3 (0.10–0.84)	
Gln/Arg	Low	1 (1.0)	8 (3.9)	0.3 (0.04–2.96)	
	High	40 (39.6)	76 (37.3)	1.3 (0.76–2.16)	
Arg/Arg	Low	1 (1.0)	1 (0.5)	8.8 (0.00-NA)	
	High	11 (10.9)	7 (3.8)	3.6 (1.27–10.49)	
*XRCC1* gene x Salt intake					0.014
Gln/Gln	Low	26 (25.7)	42 (20.8)	1.0	
	High	22 (22.0)	68 (33.7)	0.4 (0.18–1.90)	
Gln/Arg	Low	17 (17.0)	26 (12.9)	0.8 (0.37–2.06)	
	High	24 (23.7)	58 (28.8)	0.6 (0.26–1.28)	
Arg/Arg	Low	3 (2.7)	4 (1.9)	1.1 (0.20–5.56)	
	High	9 (8.9)	4 (1.9)	5.3 (1.34–21.22)	
*XRCC1* gene x Smoking					0.189
Gln/Gln	Nonsmoker	21 (20.8)	59 (29.2)	1.0	
	Smoker	27 (26.7)	51 (25.3)	2.6 (0.84–8.10)	
Gln/Arg	Nonsmoker	20 (19.8)	46 (22.7)	1.3 (0.63–2.73)	
	Smoker	21 (20.8)	38 (18.8)	2.6 (0.86–7.87)	
Arg/Arg	Nonsmoker	8 (7.9)	3 (1.5)	7.0 (0.65–29.55)	
	Smoker	4 (4.0)	5 (2.5)	3.6 (0.58–22.93)	
*XRCC1* gene x Alcohol drinking					0.380
Gln/Gln	Nondrinker	17 (16.9)	57 (28.2)	1.0	
	Drinker	31 (30.7)	53 (26.5)	2.6 (0.11–6.09)	
Gln/Arg	Nondrinker	20 (19.8)	48 (23.7)	1.5 (0.74–3.42)	
	Drinker	21 (20.8)	36 (17.8)	2.6 (0.05–6.21)	
Arg/Arg	Nondrinker	8 (7.9)	4 (1.9)	6.2 (0.63–24.23)	
	Drinker	4 (3.9)	4 (1.9)	5.7 (0.82–39.44)	
*XRCC1* gene x Pork oil intake					0.226
Gln/Gln	Low	41 (40.5)	101 (50.0)	1.0	
	High	7 (6.9)	9 (4.5)	1.9 (0.66–5.91)	
Gln/Arg	Low	38 (37.6)	77 (38.0)	1.3 (0.75–2.32)	
	High	3 (2.9)	7 (3.5)	1.2 (0.29–4.97)	
Arg/Arg	Low	10 (9.9)	6 (3.0)	3.9 (0.29–12.27)	
	High	2 (2.2)	2 (1.0)	3.7 (0.47–29.29)	

*ORadj.*: adjusted odd ratio, *95% CI*: 95% confidence interval using conditional logistic regression, *p*-value from interaction assessment, were adjusted for gender and age, *NA*: not applicable

## Discussion

Our objective was to investigate effect of environmental risk factors and the *XRCC1* gene and how they related to the incidence of stomach cancer. This study found that there was an interaction effect between Arg/Arg homozygosity and high salt or vegetable oil intake leading to increased susceptibility to stomach cancer compared to other *XRCC1* genotypes. That is, the *XRCC1* genotype modifies the impact of high dietary salt and vegetable oils on the risk of stomach cancer.

Several studies have demonstrated that factors such as gender, smoking, alcohol use and *H. pylori* infection enhance the risk of stomach cancer for some *XRCC1* genotypes, but not for others [24, 25].This is inconsistent with our study, although we found that smoking and alcohol consumption modify the effect of *XRCC1* gene on the risk of stomach cancer in our sample, we could not demonstrate these effects to be statistically significant. Previous studies have found that high consumption of vegetable oil, saturated fat and cholesterol increased the risk of stomach cancer [9–11]. Numerous studies have also reported on the risk of salt intake and its association with stomach cancer [2, 4, 6–8]. However, no study has established differential stomach cancer risks of salt and fat intake for different *XRCC1* genotypes. We demonstrate that high fat and salt intake are particularly risky for the *XRCC1* Arg/Arg genotype, and importantly, these environmental factors could not be shown to be associated with increased risk of stomach cancer in the Gln/Arg or Gln/Gln XRCC1 genotypes.

Our study demonstrates that Thai people (typically of Thai-Lao ethnicity) are likely to be genetically susceptible to the stomach cancer risk factors of high vegetable oil and salt intake. Our results differ from studies conducted in western countries, which shown either different environmental risk and/or gene-environment interactions. For instance, a study conducted in Poland found that *XRCC1*, *XPD* and *MGMT* polymorphisms modified the magnitude of risk associated with low intake of fruits or vegetables and smoking for gastric cancer. [24] A Brazilian study revealed the interaction between of *XRCC1* 399Gln and *XRCC3* 241Met with gender, smoking, alcohol consumption and *H. pylori* infection in terms of gastric cancer. [25] These differences in results may reflect differences in gene-environment interaction across these populations of different ethnicity. However, difference between the present study and the findings of others is perhaps more likely to stem from differences in gene and environmental risk factors considered, Or a reflection of study design.

In summary, this study shows a significant effect of high fat and salt intake and the *XRCC1* gene as risk factors for stomach cancer. However, while smoking, alcohol consumption and pork oil intake were associated with stomach cancer in our sample, the magnitude of these effects were not strong enough to attain statistical significance. Hence, our results may have policy implications in the sense that civic education and awareness of the results should be provided and aimed at Thailand as a whole, but it will be necessary to confirm these findings with a larger sample size before giving serious consideration to any interventions.

There were several limitations in the present study. First, our sample size was relatively modest, and comprised of a comparatively ethnically homogenous sample of the north-eastern Thai population. Whether the associations we demonstrate, especially differential risk associated with high vegetable oil and salt intake across genotypes, holds for populations of other or mixed ethnicity is an important question that still remains. Future studies involving other populations need to be conducted to determine if certain *XRCC1* genotypes along with vegetable oil and salt intake pose a risk of stomach cancer in those populations. A second limitation is that even though *H. pylori* has been previously identified as an important risk factor in the development of stomach cancer, we only had patient history of *H. pylori* exposure in our stomach cancer cases, but had no such information for our control participants. This made it impossible to examine the impact of *H. pylori* as an independent risk factor, or indeed, whether *H. pylori* exposure confounds or modifies the *XRCC1* genotype effect, or the impact of elevated vegetable oil or salt intake. The strengths of the present study were that it was a hospital-based matched cases-control study made up of all newly diagnosed cases of stomach cancer, which were confirmed by histopathology. Furthermore, controls were matched for age, gender and admitted at the same time and in the same ward as cancer cases. All data collection was conducted by expert-trained nurses. The laboratory investigating the *XRCC1* gene used the real-time PCR-HRM technique and conditional logistic regression for data analysis.

## Conclusions

In conclusion, the effect of the *XRCC1* gene homozygosity, particularly Arg/Arg, on the risk for stomach cancer was elevated by a high intake of vegetable oils and salt.

## Abbreviations

°C: Celsius
95% CI: 95% confidence interval
Arg: Arginine
DNA: Deoxyribonucleic acid
Gln: Glutamine
HRM: High resolution melting
OR _adj_: Adjusted odds ratios
OR _c_: Crude odds ratios
PCR: Polymerase chain reaction
SD: Standard derivation
*XRCC1*: X-ray repair cross-complementing group 1
